# Was *Lates* Late? A Null Model for the Nile Perch Boom in Lake Victoria

**DOI:** 10.1371/journal.pone.0076847

**Published:** 2013-10-18

**Authors:** Andrea S. Downing, Nika Galic, Kees P. C. Goudswaard, Egbert H. van Nes, Marten Scheffer, Frans Witte, Wolf M. Mooij

**Affiliations:** 1 Aquatic Ecology and Water Quality Management Group, Department of Environmental Sciences, Wageningen University, Wageningen, The Netherlands; 2 Department of Ecology, Environment and Plant Sciences, Stockholm University, Stockholm, Sweden; 3 Institute for Marine Resource and Ecosystem Studies (IMARES), Wageningen UR, Yerseke, The Netherlands; 4 Institute of Biology, Leiden University, Leiden, The Netherlands; 5 Department of Aquatic Ecology, Netherlands Institute of Ecology (NIOO), Wageningen, The Netherlands; North Carolina State University, United States of America

## Abstract

Nile perch (*Lates niloticus*) suddenly invaded Lake Victoria between 1979 and 1987, 25 years after its introduction in the Ugandan side of the lake. Nile perch then replaced the native fish diversity and irreversibly altered the ecosystem and its role to lakeshore societies: it is now a prised export product that supports millions of livelihoods. The delay in the Nile perch boom led to a hunt for triggers of the sudden boom and generated several hypotheses regarding its growth at low abundances – all hypotheses having important implications for the management of Nile perch stocks. We use logistic growth as a parsimonious null model to predict when the Nile perch invasion should have been expected, given its growth rate, initial stock size and introduction year. We find the first exponential growth phase can explain the timing of the perch boom at the scale of Lake Victoria, suggesting that complex mechanisms are not necessary to explain the Nile perch invasion or its timing. However, the boom started in Kenya before Uganda, indicating perhaps that Allee effects act at smaller scales than that of the whole Lake. The Nile perch invasion of other lakes indicates that habitat differences may also have an effect on invasion success. Our results suggest there is probably no single management strategy applicable to the whole lake that would lead to both efficient and sustainable exploitation of its resources.

## Introduction

*“The trigger for the Nile perch irruptions is not known; it is interesting and mysterious that the fish should have persisted for so long and at such low densities before the explosion.”* [1]

Nile perch were first introduced surreptitiously to the Ugandan side of Lake Victoria in 1954 after a decline in native tilapia and other major food fishes [2], [3]. While a few Nile perch individuals were sighted over the years, this introduction did not initially cause any apparent changes to the system. Nine years after the unofficial introductions (1963), authorities in Uganda and Kenya carried out official introductions of both adult and fingerling Nile perch. Twenty years after this, the first major catches of Nile perch were reported in Uganda, and over the course of four years, between 1982 and 1985, Nile perch had replaced most indigenous species (mainly haplochromine cichlids) in catches. The same occurred a few years later in Tanzania, at the opposite end of the lake, between 1983 and 1987 (table 1) [2], [3].

**Table 1:** How the Nile perch was introduced and invaded, from Goudswaard *et al*. (2008).

Year	Event	Location
1954	Illegal introduction of unknown number (& size) of Nile perch	Jinja, Uganda
1960	Catch of 8 Nile perch between 28–43 cm long	Jinja, Uganda
May 1962–September 1963	Official introduction of 35 subadults (16–43 cm) and 339 fingerling Nile perch	Entebbe, Uganda
1963	Official introduction of 8 individuals (size unknown)	Nyanza Gulf, Kenya
1979–1982–1983	Onset of Nile perch boom	Kenya- Uganda- Tanzania
Until 1985	Catches of adult and subadult Nile perch	Mwanza, Tanzania
1986–1985–1987	Peak of Nile perch boom	Kenya- Uganda- Tanzania
1981–1985	First wave of Nile perch boom	Kenya-Uganda
1983–1987	Final wave of Nile perch boom	Tanzania

The sudden Nile perch invasion and haplochromine collapse caused major social and economic changes in lakeside populations [4]–[6]. The fisheries of native species used to provide only for a local and regional market and were managed on small scale- and value-investments. Nile perch, however, are exported internationally and processed at a larger scale (in filleting factories) [4]. To allow factories and export businesses to operate at capacity requires maintaining constant high catches of Nile perch. Since the mid-1990s however, Nile perch catches have been fluctuating, and some haplochromine species have increased in abundance [7], [8]. Uncertainty in catches and the resurgence of haplochromine species have been interpreted – though not unanimously – as indicative that Nile perch stocks are being overfished [9]–[11]. A key consideration when determining maximum sustainable yields of a stock lies in understanding the behaviour of the fished population at low densities.

A common question among lake Victoria scientists is why did it wait 25 years after its introduction to invade [1], [12]? Also, while Nile perch successfully invaded and became dominant in lakes Victoria, Kyoga and Nabugabo – where they were introduced – they are only found in low densities in lakes Chad, Turkana, Albert and the man-made Lake Volta [13]–[16] – where they are native. Therefore, the Nile perch 25-year invasion delay, compounded with its cannibalistic tendencies and the fact that it has not colonised all environments with equal success, have led to several hypotheses regarding the viability of the stock below a certain density.

Kitchell *et al*. (1997) performed a modelling study aimed at understanding the balance between fishing pressure on Nile perch and predation pressure of Nile perch on haplochromines. While identifying the caveats of their modelling approach, the authors stated that increased fishing pressure might reduce cannibalism and have a compensatory, i.e. beneficial, effect on recruitment of juveniles to the adult stage, implying that growth rates of the population would be higher at low population densities.

A later ecosystem study [18] came to the opposite conclusion: assuming haplochromines are competitors or predators of juvenile Nile perch, increased fishing pressure on Nile perch would release predation pressure on haplochromines and lead to an increase in their abundance, which in turn would lead to a decrease in Nile perch recruitment. This scenario would imply that growth rates of the population would decrease below a certain Nile perch population density: the depensatory effect of haplochromines on recruitment would create an Allee effect. The strength of this hypothesis was later reinforced by the observation that the depensation effect might even have slowed down the invasion of Nile perch, and that the Nile perch boom was only made possible by the prior decline in haplochromine abundance [19].

Goudswaard *et al*. (2008) state that the 25 year time-span between the introduction and invasion is remarkable, as is the fact that the Nile perch invasion appears to have started in Kenya rather than in Uganda, where they were first introduced. They hypothesize that depensation by haplochromines might be behind this pattern; indeed, haplochromine stocks collapsed first in Kenya, and might thus have allowed for Nile perch to boom [2].

A recent comparative study of Nile perch diets in the Mwanza Gulf (Tanzania) before and after the resurgence of haplochromines [20] indicates that haplochromines are the preferred prey of Nile perch and that cannibalism mostly occurs when they are absent. The authors hypothesise that the return of haplochromines will therefore either compensate for the negative effects of cannibalism or – if they have no depensation effect – allow for an increase in Nile perch stocks.

**Figure 1:**
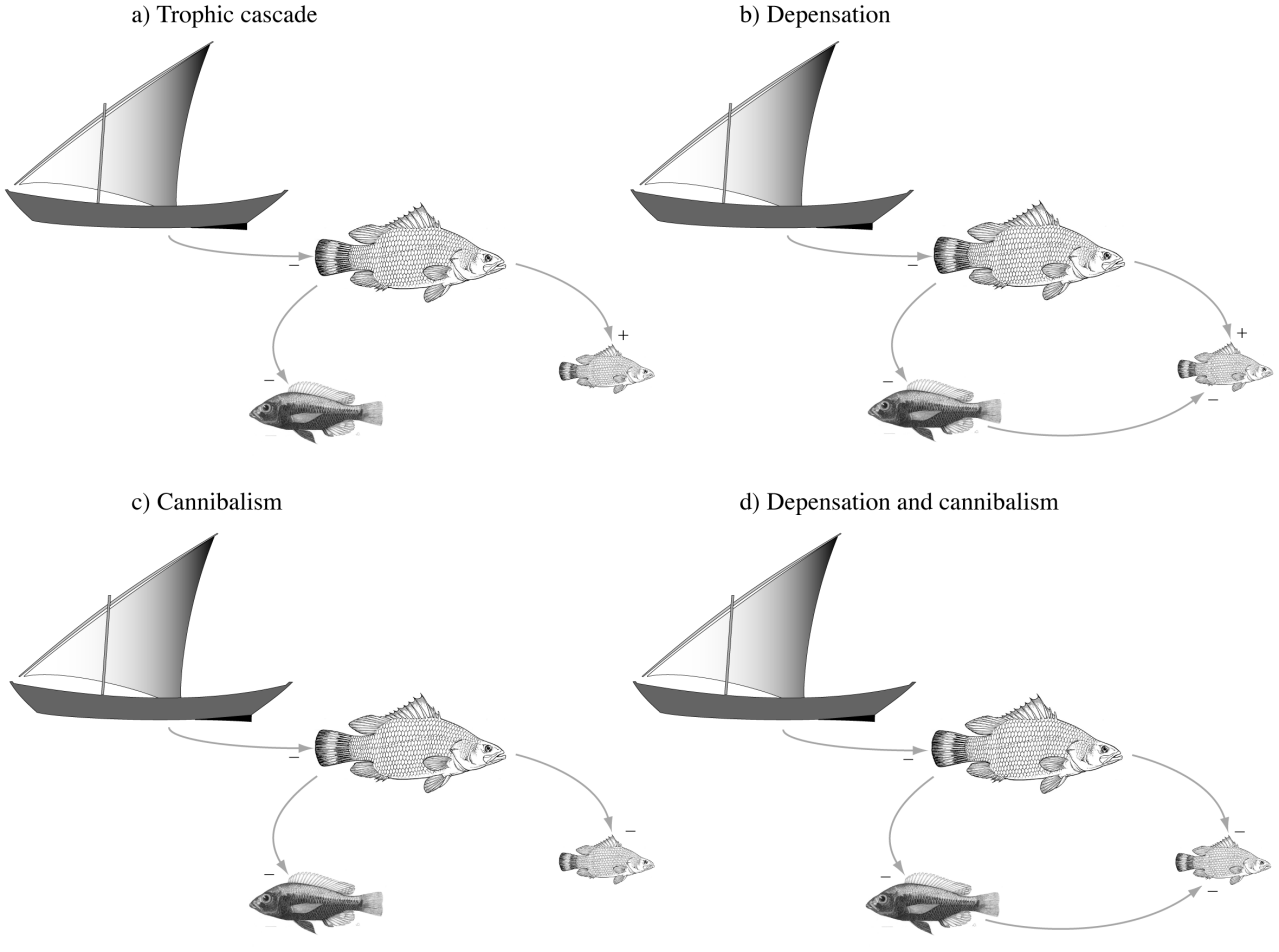
The different stock-recruitment relationships hypothesised for Nile perch. The top and right-hand fish of each panel represent Nile perch adult and young, respectively, the left hand fish represents haplochromines. a) There is no negative effect on recruitment; b) haplochromines have a negative effect; c) Nile perch have a negative effect; d) both haplochromines and Nile perch have a negative effect. To read the effect of any single component of each diagram on another it is connected to, follow arrows and multiply the signs: e.g. in d) the effect of fishing, via haplochromines on juvenile Nile perch is overall negative (−*−*−), whereas the effects of fishing, via large Nile perch, on juveniles is positive (−*−): they here cancel each-other out. In b) however, fishing has a negative effect following both pathways (−*−*− vs. −*+).

Based on these hypotheses, we can draw four alternative stock-recruitment relationships for Nile perch in Lake Victoria: a) Recruitment is a function of stock-size, there is no cannibalism or depensatory effect; b) Haplochromines have a depensatory effect on Nile perch recruitment; c) In the absence of haplochromines, Nile perch have a negative effect on their own recruitment through cannibalism; d) There is an alternation of depensation by haplochromines and cannibalism on recruitment (fig. 1).

Each of these descriptions implies a different possible prediction about the future of Nile perch stocks, and can lead to very contrasting optimal actions for the management of Nile perch (table 2). For example, if haplochromines have a strong depensatory effect, the system could have alternative stable states, where either Nile perch or haplochromines are dominant but do not co-exist in high abundances [21]. In such a case, the return of haplochromines indicates that fishing pressure on Nile perch should be reduced, or else stocks might collapse. If on the other hand Nile perch exerts a negative effect on its recruits, fishing pressure on Nile perch should probably be managed so as to best maintain an abundant enough stock of haplochromines in the system – as hypothesized by Kitchell *et al*. (1997), or Nile perch might undergo strong population cycles [22].

**Table 2:** The effects of different types of stock recruitment relationships (c.f. fig.1) on the introduction, settlement and removal of Nile perch, with theoretical management possibilities for the maintenance of sustainable Nile perch stocks. Np =  Nile perch, H = haplochromines.

Stock-recruitment relation	Description	Introduction of Nile perch	Established Nile perch	Fishing Nile perch	Theoretical management options
a) Trophic cascade	Np have a negative effect on H and a positive effect on their own recruitment	Np eats its way into the top position of the food web.	High abundance of Np maintains low abundances of H.	Lower abundance of Np lead to increase in H. Np recruitment declines with adult stock.	Fish less
b) Depensation	Np have a negative effect on H and a positive effect on own recruitment. H have a negative effect on Np recruitment.	Too high an abundance of H might prevent or slow the establishment of Np.	High abundance of Np maintains low abundances of H.	Lower abundance of Np lead to their sudden collapse and the dominance of H.	Cull H. Introduce more Np.
c) Cannibalism	Np have a negative effect on H and on own recruitment.	Np establishment proceeds as normal in the presence of H, may be slowed by cannibalism in the absence of H.	High abundance of Np maintains low abundances of H. Np dominated by large individuals and recruitment is low.	Lower abundance of Np lead to more recruitment and increase in H abundance. Np population is stunted	Fish less. Shift fishing effort from large to smaller Np.
d) Depensation & cannibalism	Np have a negative effect on H and on own recruitment. H have a negative effect on Np recruitment.	Too high an abundance of H might prevent or slow the establishment of Np. Too low an abundance of H might slow the establishment of Np.	High abundance of Np maintains low abundances of H. Np dominated by large individuals and recruitment is low.	Lower abundance of Np leads to less cannibalism but more depensation. Np recruitment declines with adult stock.	Fish less.

Recruitment interferences can produce Allee effects and are most apparent at low population densities [23]: their presence is therefore difficult to identify while the Nile perch population is well established. We use the limited information there is on the Nile perch introduction to estimate when its upsurge should have been expected, and compare this with available data, to identify and discuss the importance of various stock-recruitment interferences.

Then, for lack of better knowledge on Nile perch migration, we assume Nile perch dispersed through the lake, and investigate what minimum requirements would have been for Nile perch to disperse across the lake in the time observed. We use these results for a broader discussion on Nile perch migration and as a theoretical baseline to which further research on Nile migration can be compared.

## Methods

### Data

Goudswaard *et al*. (2008) published the most consistent compilation of data on the Nile perch introduction and invasion, gathering and standardising data from trawl surveys that had been carried out with different vessels, engines, and net sizes. Even though these data are not necessarily representative of the actual total biomass present in the lake – because different mesh-sizes are differently selective of fish-sizes – the standardisation by Goudswaard *et al*. (2008) makes them comparable, at least in terms of the timing of the observed changes. For each country or area surveyed, the authors arbitrarily define the onset of the boom as the moment when the density first reached 45 kg per hour of trawl (table 1).

The number and size of fish illegally introduced in 1954 is unknown, but was probably sufficient to produce a viable population, since eight sub-adult Nile perch were caught in 1960. These were too small to be the introduced individuals themselves and were assumed to be their progeny [2].

### Timing of the Boom

Data on the introduction and invasion of Nile perch in Lake Victoria are quite few and scattered and produce no insight into the mechanisms or biology behind the invasion process. For this reason, we use a model of precision equal to those data, i.e. the logistic growth model, and with independent estimates of growth and carrying capacity, measure how many years after its introduction to Lake Victoria Nile perch growth would have reached its upsurge phase, and how soon thereafter it would slow down and reach carrying capacity. We compare these two metrics – the time of onset of the boom and time to carrying capacity – with the observed time of the Nile perch boom and the year of the highest catch as compiled by Goudswaard *et al*. (2008).

In our model equation (Eq. 1), *N* represents Nile perch abundance (t/km^2^), *r* the annual growth rate (yr^−1^) and *K* the population carrying capacity (t/km^2^).
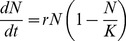
(1)


We used a growth rate of 0.73(yr^−1^) derived by [24]. In their study, the authors use a physiologically structured population model fitted to Nile perch to find how resource abundances and the length at which they shift diets influence the growth rate. 0.73(yr^−1^) represents the maximum growth rate under an unlimited mixed resource and a wide range of lengths at diet shift. We use a carrying capacity of 963200 tonnes for the whole lake, from a density of 14 (t/km^2^) derived from Pitcher and Bundy [33] multiplied over the surface area of the lake (68,800 km^2^) and set the initial “inoculum” (N_0_) to four 20 cm-long Nile perch. Using equation 1 we calculate biomass growth over 35 years and identify the year where biomass first exceeds 5% of carrying capacity (approximately same density threshold as defined by Goudswaard *et al*. [2]) as the year of the onset of the boom, and the year where biomass first exceeds 95% of carrying capacity as the peak of the boom.

We then evaluate how mild depensation would affect the timing of the Nile perch invasion. We do so by setting a lower growth rate (90%, 80%, 70% and 50% of 0.73) until Nile perch reaches the 50%, 100% or 200% of the biomass used as our indicator of the onset of the boom. Furthermore we performed a semi-analytical sensitivity analysis to find which parameters determine the onset of the boom (see File S1).

### Dispersal

Because the Nile perch boom did not take place over the whole lake simultaneously, we assume that Lake Victoria is larger than the homogeneous-distribution area of Nile perch, i.e. the area Nile perch would cover without dispersing, over which its density would increase homogeneously. Therefore, to describe the migration or dispersal of Nile perch across Lake Victoria, we need two parameters: firstly, one that reflects density-dependence – or the homogeneous-distribution area of Nile perch – that influences the threshold at which a population growth solely serves to increase population density to serving both as an increase in density and to expand over space; and secondly, a migration or dispersal rate, that sets a balance between energy allocation to growth and to migration.

There is, to our best knowledge, no information on either the density-dependence of migration or on dispersal rates of Nile perch. A preliminary migration study carried out in the late 1980s found that Nile perch can swim 50 km in a single week, and up to 150 km in 6 months: an individual can thus cross the lake within a year [34]. We therefore assume that there are no physiological boundaries to Nile perch dispersal, and instead focus our attention to effects of basic triggers of dispersal and use a parsimonious model.

We first investigate the effects of density-dependence by testing different territoriality scenarios and assume that Nile perch disperse according to their density: a highly territorial Nile perch would have a small free-distribution range and start moving at low population densities and a less-territorial Nile perch would have a large free-distribution range and start dispersing later. We divide the lake into different numbers of cells *n* of equal area to test the effects of territoriality: many small cells to fit a high-territoriality scenario and fewer large cells to represent a low-territoriality scenario. We create a one-dimensional model, where we assume the cells are distributed linearly, with the two extremities representing the most northern and southern parts of the lake and Nile perch diffuses in one dimension (*i*)(Eq 2). We also build a 2-dimensional lattice-model, where Nile perch can diffuse in two directions (*i* and *j*)(Eq. 3). We fit a modified logistic growth model to each cell that includes migration to and from neighbouring cells (*i*−1; *j*−1 and *i*+1; *j*+1) at a migration rate *m* (yr^−1^). We include border conditions, so that biomass cannot leave the lattice.
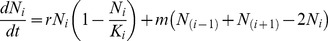
(2)

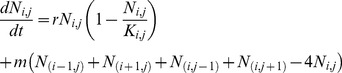
(3)


The dispersal rate *m* is a function of the number of areas the lake is subdivided into (*n*). For every value of *n* tested (*n_i_* = 2; 3; 4; 5; 10; 20 and *n_i,j_* = 5,5; 10,10; 20,20), we calibrate *m* so that the onset of the boom in the first and last cells fit observations for Kenya and Tanzania respectively.

## Results

### Timing of the Nile Perch Boom

**Figure 2:**
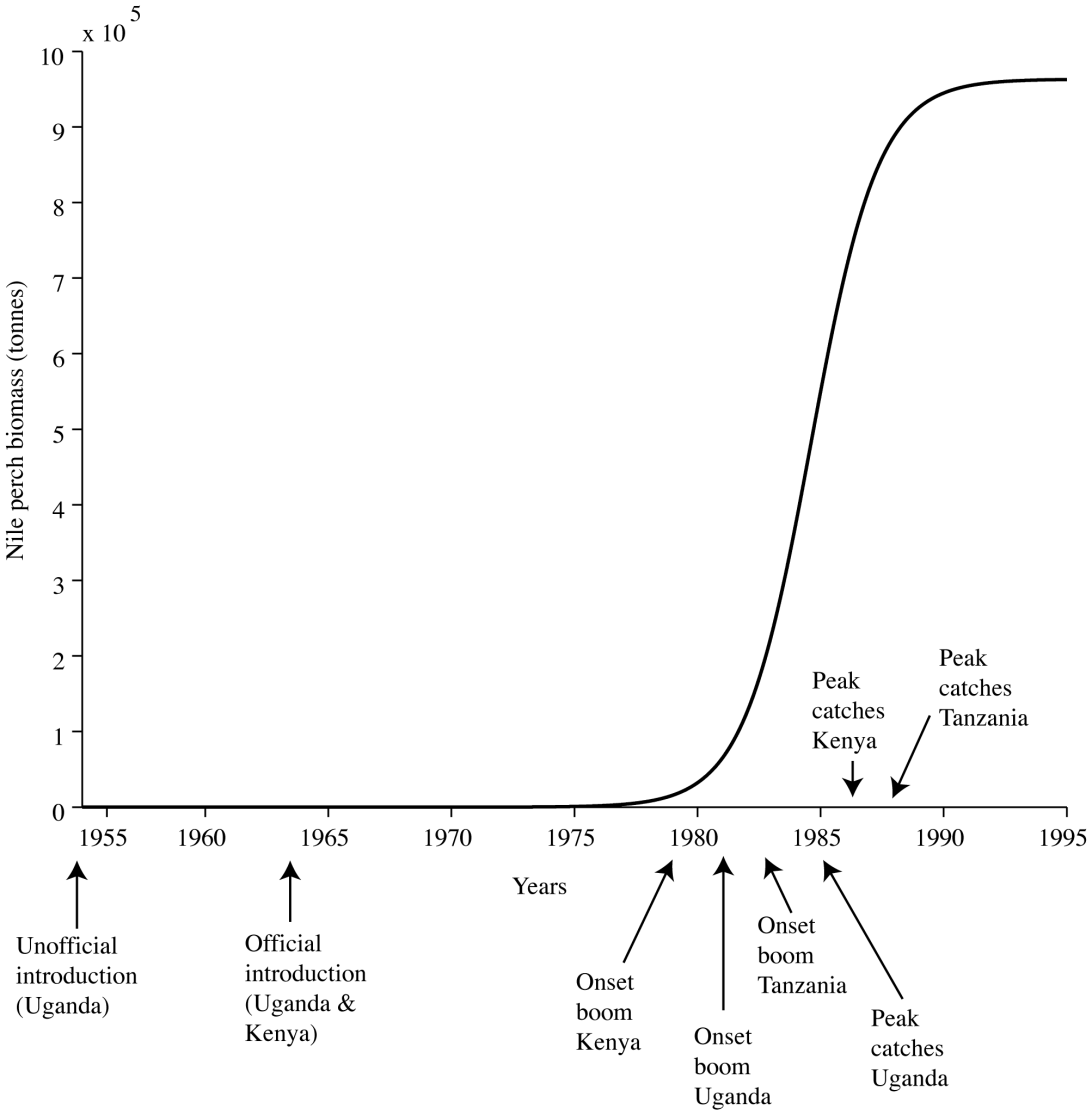
The logistic curve, with N_0_ = 0.00039 tonnes, r = 0.73 year^−1^; K = 963200 tonnes. The onset of the boom phase matches observations of the timing of the Nile perch boom.

Our logistic growth model yields an onset of the boom 25 years after the introduction, with a carrying capacity reached 8 years later. This corresponds to the onset of the Nile perch boom taking place in 1979, given the observation that the initial illegal stocking of Nile perch carried out in 1954 actually took seed and produced a viable population (fig. 2). This finding is robust to whether the later official introduction was successful or not: indeed, by 1963, the population introduced in 1954 would already have grown to produce a biomass twentyfold larger than that of the 35 adults and 339 fingerlings introduced in 1963. The later official introductions therefore represent a “drop in the Nile perch sea” and have no effect on the timing of the Nile perch boom.

Even though there is a lot of uncertainty in the initial 1954 stocking size (*N_0_*), we find the assumption that a few sub-adults constituted the initial introduction realistic, considering that it is probably not larger than the official introduction that occurred later in Uganda for example. The timing of the boom is mostly dependent on the growth rate (Supporting information S1), and a change in the initial stocking size has an effect 20 times smaller than that of the growth rate. Indeed, we find that doubling the initial population size to 10 individuals only leads to the onset of the boom occurring one year earlier than expected. A smaller initial population-size leads to the peak of the Nile perch-boom taking place one year later. An initial stock-size of 4 subadults produce a population larger than the catches of 1960, as well as a timing for the onset and peak of the boom that matches observations. The growth rate we use (0.73 yr^−1^), derived from Downing *et al.* (2013) is realistic and close to the average over all age-classes derived by Kitchell *et al.* (1997) (between 0.6 yr^−1^ for adults and 0.8 yr^−1^ for the youngest cohort) [17]. Though the elasticity analysis illustrates how sensitive the results are to *r*, a growth rate of 0.8 yr^−1^ – which is a high estimate of growth rates for large fish such as Nile perch – would only yield a difference of two years on the onset of the boom, probably not enough to change the perception of a delayed invasion.

Depensation, whereby growth of Nile perch would have been lower than maximum at low population densities, would delay the Nile perch boom, even if the depensatory effect has a very low biomass threshold (table 3). Indeed, the result is much more sensitive to the strength of the depensation than to the duration of the depensatory effect, indicating that any strong effect on growth early on in the invasion process would probably have delayed the Nile perch invasion.

**Table 3:** Effects of depensation on the year of onset and peak of the Nile perch boom.

		Depensation effect (% r)						
		100%		90%		80%	70%	50%
Depensation threshold (%B_ini_ t)	50%	1979	1982		1985		1989	2003
		1987	1990		1993		1998	2012
	100%	**1979**	1982		1985		1990	2004
		**1987**	1990		1994		1998	2013
	200%	1979	1982		1984		1990	2004
		1987	1990		1994		1999	2014

We illustrate depensation as a lower population growth rate when the Nile perch population is at low densities. The upper value in each cell represents the calculated onset of boom under each depensation scenario, and the lower value represents the peak of the boom. Depensation tests are read as follows: in columns – reduced growth rate as a percentage of 0.73. In rows: Nile perch biomass until which depensation is effective (arbitrarily centred around the indicator of the onset of the boom B_ini_) (rows) on the timing of the start and peak of the Nile perch boom (top and lower year in each cell, respectively), in bold, the standard, no-depensation situation.

### Nile Perch Migration Wave

**Figure 3:**
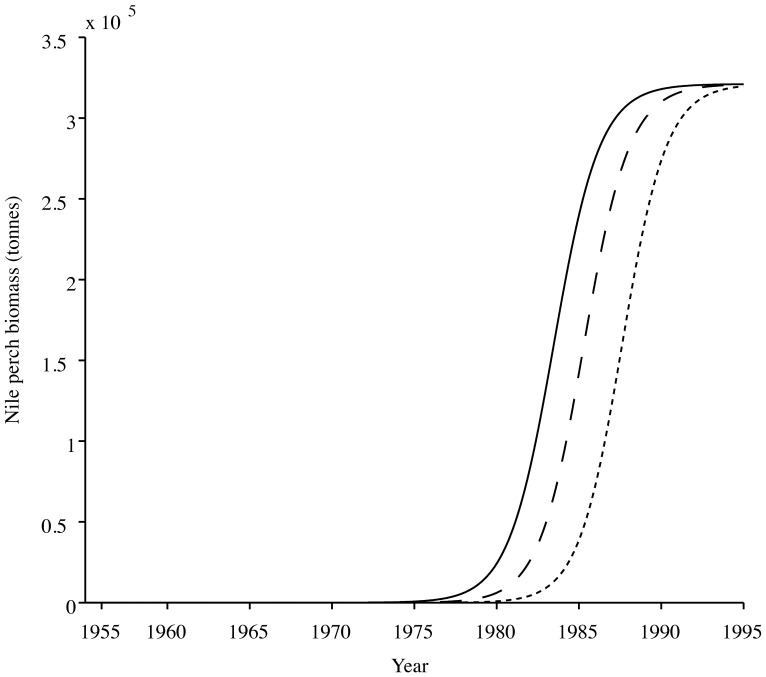
Nile perch dispersal wave with onset in the north in 1979 and in the south in 1984 (n = 3 and m = 0.11). The solid line represents the first wave in the north of the lake, the dashed one in the second segment of the lake, and the dotted line represents the final wave in the southernmost part of the lake. Adding resolution to the dispersal process (i.e. increasing n), would require an increase in *m*, which delays the dispersal wave in the north and speeds it up in the south.

**Figure 4:**
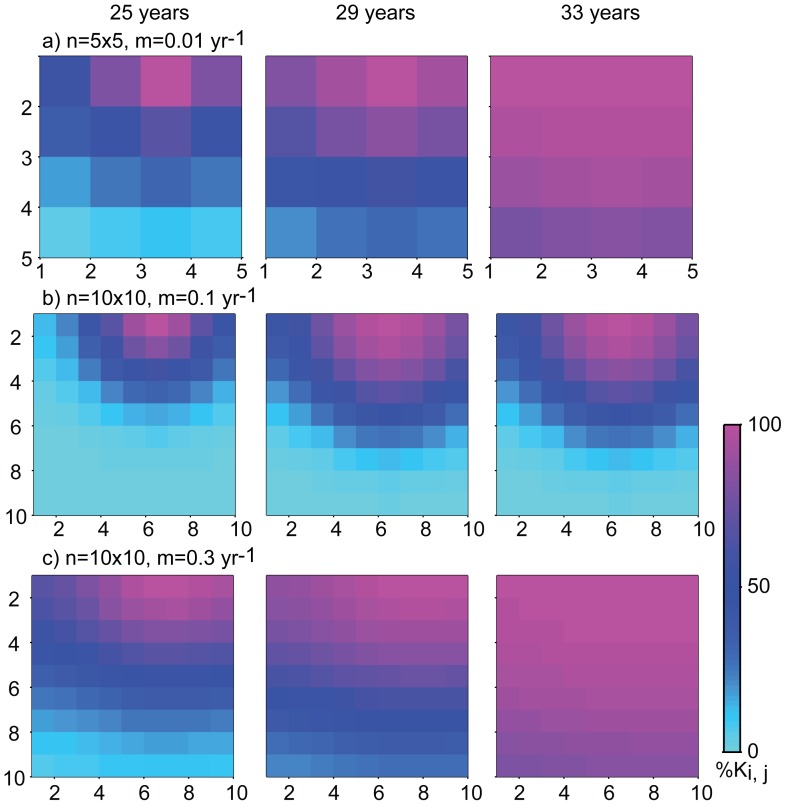
Nile perch dispersal in two dimensions. If the number of cells is small (e.g. under a) a small dispersal rate *m* can produce a colonisation wave, where the expansion starts after 25 years, and the lake fills in 33 years. As the number of cells increase (b and c), representing a more territorial behaviour of Nile perch, a high dispersal rate is necessary for a colonisation of the lake in 33 years, However, then the dispersal is also more homogeneous (c versus b).

Nile perch did not initially distribute themselves homogeneously over the lake, instead they invaded the lake as a wave, starting in the north and reaching the south a few years later. In the absence of independent dispersal estimates, we adjusted our model’s dispersal parameter *m* so that is was sufficiently large to bring enough individuals across the lake, and for the boom to occur there in 1985, and small enough not to dilute the first cell too much and delay the first boom (fig. 3). The value of *m* is dependent on the size of each cell: the more cells we divide the lake into, the larger *m* needs to be for the migration wave to reach the last cell, and that this should occur within the time limit. Therefore, our one-dimensional model can only reproduce the migration wave for a number of cells smaller than 4. In a two dimensional model, where biomass can diffuse in four directions, the migration only takes place as a wave at lower values of *m*, and thus for a low resolution in term of number of cells. At higher dispersal values, the migration occurs homogeneously over the lake (fig. 4). Comparing observations of a migration wave going through the lake with our lattice model, we might suggest the Nile perch has quite a large territory. Should Nile perch be found to be a very territorial fish, we could only reproduce the observed migration wave without altering results for the timing of the boom by increasing the carrying capacity of the system.

## Discussion

### The Timing of the Nile Perch Boom

With independently determined values for growth, carrying capacity and reasonable estimates for the biomass of Nile perch initially introduced, our logistic model yields an onset of the boom and time to carrying capacity that match the coarse observations of the Nile perch boom in Lake Victoria. Therefore, the timing and speed of the Nile perch invasion in Lake Victoria should *not* appear surprising or remarkable. Half a kilo of unhindered Nile perch takes 33 years to fully invade Lake Victoria (1954–1987), due to a constant exponential growth that becomes apparent only in its latest stages, with no external triggers for the boom necessary.

Furthermore, the Nile perch stock measures of Kudhongania and Cordone (1974) broadly fit within the predictions of the model. They estimated that the standing stock of Nile perch in 1969–1970 was 402 tonnes, through conversion of trawl data by the swept area method [26]; our logistic growth model indicates the population would have crossed the 400 tonnes threshold in 1972. Given the level of approximation in the data conversion method and the uncertainty as to the number of individuals initially introduced, we find this is a reasonably nice fit to our model.

A population growth model with three key parameters fits the available data and simple logistic growth is therefore the most parsimonious explanation behind the timing of the Nile perch invasion. These results rely on uncertain data – we do not know how many or what age Nile perch were initially introduced in 1954 nor if individuals have a significantly different growth rate at introduction. However, the findings are robust to the uncertainty in both stocking-size and growth rate, as variations within the uncertainty range would only change results by a couple of years – which fits the uncertainty in the observations and would probably not have affected the common perception of the delay in the Nile perch boom. To summarise: the parameter settings are realistic, the results are reasonably robust and leave us no reason to conclude differently than that the Nile perch invasion at the scale of the whole lake occurred as should have been expected as it entered its new resource-rich environment.

### The Migration Process

The Nile perch boom took place first in the north of the lake – though interestingly in Kenya before Uganda – where it was introduced and then crossed the lake as a wave or front, reaching the southernmost part three years later [2]. This indicates that Nile perch did not disperse completely freely and therewith increase in abundance homogeneously over the whole lake, but migrated from one end to the next. To reproduce this migration with a simple linear dispersal model, we must assume that Nile perch are not very territorial and have a broad dispersal range. Should further research on Nile perch migration and spatial behaviour find that Nile perch has a low dispersal range, that there are effective barriers to its distribution or that Nile perch has territorial behaviour, the model would need to be adapted; either an increase in the carrying capacity of the lake or a more complex expression of its migration would be necessary to illustrate the migration process without affecting the timing of the Nile perch boom at the scale of the whole lake.

### Further Considerations

Our results indicate that a prior decline in haplochromine abundance was not a prerequisite for the Nile perch invasion. Nonetheless, they do not exclude the possibility that negative interactions – such as depensation or cannibalism – influence Nile perch growth. Our results here suggest that such processes did not have any significant effect on the timing of the Nile perch invasion on the large temporal and spatial scales that reflect the resolution of the observations on the Nile perch invasion in Lake Victoria. However, Goudswaard *et al*. (2008) point out that the Nile perch boom started in Kenya before Uganda, and match this observation with the decline of haplochromines that appears to have taken place in Kenya first [2]. While we find that the timing for the upsurge in Kenya could have been expected, perhaps some mechanism did delay the invasion from happening first in Uganda. Our findings provide us with a new perspective from which to look at the mystery of the Nile perch invasion; indeed, instead of asking what triggered the onset of the boom in Kenya, we might now ask what delayed the boom in Uganda.

Lake Victoria is the world’s second largest lake, and it constitutes a very heterogeneous assemblage of habitats that contain different combinations of species (i.e. different foodwebs). Therefore, processes such as depensation and cannibalism as well as fishing-pressure and the compensatory effects of eutrophication (through increased productivity in lower trophic levels) are probably not homogeneously distributed over the lake; each of these processes might very well influence Nile perch growth and dynamics at a smaller foodweb or habitat scale. Growth-influencing processes might be seasonal and cancel each other out over the year; Nile perch distributions vary seasonally and geographically [10] and therefore, a strong depensation-season might be followed by a high productivity and growth season. Also at larger time scales, one could hypothesize that eutrophication in the lake might have produced more resources for juvenile Nile perch and compensated for the depensatory effects of haplochromines. We here find that there was no prevalent effect of any such process on the Lake Victoria-scale, but do not dismiss the fact that these processes occur at smaller scales, or in different areas.

In similar ways to Lake Victoria, Nile perch were introduced to lakes Kyoga (in 1955) [3], [28] and Nabugabo (in 1960) [27], where they successfully invaded the system and depleted native haplochromine stocks [13], [14]. In Lake Kyoga however, Nile perch were caught lake-wide already in 1962– only seven years after their introduction [27], and catch reports indicate the boom occurred between 1963 and 1968 [31]. Using our model (*r* = 0.73, *K* = 24080, *N_0_* = 147 individuals [3] of unknown length – we assume 30 cm) we would have expected a Nile perch boom to occur between 1968–1976: at least five years later than actually observed. The speediness of the Nile perch invasion in Lake Kyoga might be related to the maturity of the individuals introduced, or to higher productivity of this environment. Lake Kyoga is a lot shallower than Lake Victoria, as it has an average depth of six meters, whereas Lake Victoria has an average depth of 40 meters. This might have played a role, perhaps allowing for more efficient predation, reproduction and establishment. In an interesting parallel, Kudhongania and Cordone’s 1969–1970 survey of Lake Victoria found no Nile perch beyond 29 meters of depth [25]: the Nile perch shifted to deeper waters in the 1980s [32].

In the case of Lake Nabugabo, there are no data illustrating how and when the invasion took place, all that is known is that by 1991 Nile perch was already dominant in the lake’s open waters and most native species had either disappeared or greatly declined in abundance [29], [30]. The case of Lake Nabugabo is nonetheless interesting in that it nicely illustrates of the role of different habitats on the success of Nile perch invasions. Indeed, Nile perch invaded the open waters of Lake Nabugabo, but not the wetland and swamp habitats that thus provided a refugium for some native cichlids and lungfish [30].

Despite their success in lakes Victoria, Nabugabo and Kyoga, Nile perch do not dominate all the lakes in which they are found: in lakes Albert, Chad, Turkana and the man-made lake Volta for example, they co-exist at low abundances with tilapia, haplochromines or other species [13], [15], [16]. Nile perch are therefore not immune to environmental factors or interspecific interactions that can limit their growth. It should also be noted that Nile perch are natives of lakes Albert, Chad and Turkana, as well as of the River Volta; it is likely Nile perch and other species in these water bodies have a long history of co-evolution, a history that probably should not be forgotten when considering the future of Nile perch stocks in Lake Victoria.

### Conclusions

Simple logistic growth suffices to explain the timing of the Nile perch boom and fits the principle of parsimony (Ockham’s razor). Testing the null hypothesis as we do here does not prove that Nile perch do not suffer depensatory or cannibalistic effects. Instead our analysis sets a new baseline from which to compare deviations in the invasion or establishment process of Nile perch and from which to better identify the mechanisms that might cause such deviations.

Furthermore, the fact that no single process dominates or influences Nile perch growth at the scale of the whole lake should be taken as an indicator that important population-dynamic driving processes probably occur at smaller time- and space- scales, e.g. seasonally or within different habitats. Therefore, further research should not operate from the *a priori* assumption that a consistent depensatory process influences Nile perch growth, and instead needs to identify the scales at which these processes have effect as well as how these scales compare to and are influenced by exploitation patterns. Importantly, any single management strategy aimed at maintaining sustainable stocks of Nile perch is probably not applicable to the lake as a whole, and would be most effective if based on dynamics observed at the scale of exploitation.

## Supporting Information

File S1 **Sensitivity Analysis.**
(DOCX)
