# Supplementary material for: Was Lates Late? A Null Model for the Nile Perch Boom in Lake Victoria
Source: PLoS One. 2013 Oct 18;8(10):e76847. doi: 10.1371/journal.pone.0076847 (PMC3800122; doi:10.1371/journal.pone.0076847)
Supplement: File S1 — Sensitivity Analysis. (DOCX) [file pone.0076847.s001.docx]

**Was *Lates* late? A null model for the Nile perch boom in Lake Victoria**

Andrea S. Downing, Nika Galic, Kees P. C. Goudswaard, Egbert H. van Nes, Marten Scheffer, Frans Witte, Wolf M. Mooij

## Supporting Information S1: Sensitivity analysis

We performed an analytical sensitivity analysis by deriving the derivatives of the solution of the logistic growth model with respect to the growth rate (*r*), the carrying capacity (*K*) and the initial stock size (*N_0_*). These derivatives can be interpreted as sensitivity coefficients.

The analytical solution of the logistic growth model is:

$$N\left( t \right)=\frac{e^{r t}K N_{0}}{\left( e^{r t}-1 \right)N_{0}+K}$$

The derivatives of this solution to the initial stock size and parameters, reflecting the sensitivity of the change in population-size to changes in initial stocking and parameteres are:

$$\frac{\delta N\left( t \right)}{\delta N_{0}}=\frac{e^{r t}K^{2}}{\left( e^{r t}N_{0}-N_{0}+K \right)^{2}}$$

$$\frac{\delta N(t)}{\delta K}=\frac{\left( e^{r t}-1 \right)e^{r t}N_{0}^{2}}{\left( e^{r t}N_{0}-N_{0}+K \right)^{2}}$$

$$\frac{\delta N(t)}{\delta r}=\frac{t e^{r t}K N_{0}\left( {K-N}_{0} \right)}{\left( e^{r t}N_{0}-N_{0}+K \right)^{2}}$$

We obtain the elasticity of the model to each parameter by scaling the sensitivity to population size.

$$E_{N_{0}}(t)=\frac{\delta N\left( t \right)}{\delta N_{0}}\frac{N_{0}}{N\left( t \right)}; E_{K}(t)=\frac{\delta N\left( t \right)}{\delta K}\frac{K}{N\left( t \right)}; E_{r}(t)=\frac{\delta N(t)}{\delta r}\frac{r}{N(t)}$$

Finally, we plot the elasticity of each parameter as a function of time so as to tell the effect of each parameter on population size as population grows (fig. S1).


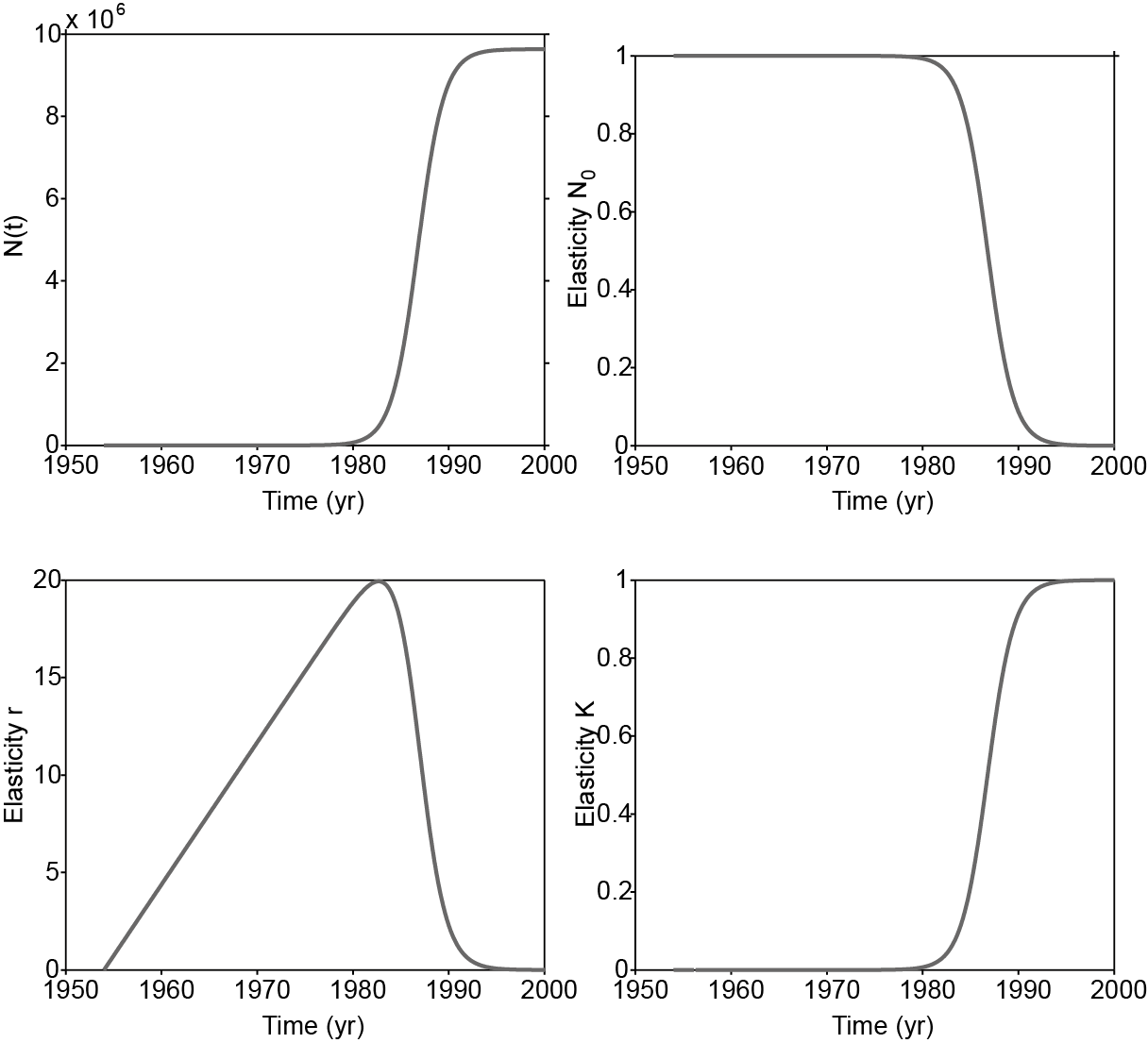


Figure S1. The sensitivity of the model to the parameters and initial conditions. (a) the original model with the default parameters (see figure 2), (b,c,d) the elasticity of resp. the initial conditions (*N_0_*), the carrying capacity (*K*) and the growth rate (*r*).

## Conclusion

The timing of the boom is almost entirely determined by the growth rate *r.* The initial stock size *N_0_* has also some impact but the exact value is relatively unimportant. The carrying capacity *K* has negligible effect on the timing of the boom, but it determines the equilibrium density.
